# Early Detection of Tuberculosis Outbreaks among the San Francisco Homeless: Trade-Offs Between Spatial Resolution and Temporal Scale

**DOI:** 10.1371/journal.pone.0001284

**Published:** 2007-12-12

**Authors:** Brandon W. Higgs, Mojdeh Mohtashemi, Jennifer Grinsdale, L. Masae Kawamura

**Affiliations:** 1 MITRE Corporation, McLean, Virginia, United States of America; 2 Department of Computer Science, Massachusetts Institute of Technology, Cambridge, Massachusetts, United States of America; 3 Tuberculosis (TB) Control Section, San Francisco Department of Public Health, San Francisco, California, United States of America; Harvard Medical School, United States of America

## Abstract

**Background:**

San Francisco has the highest rate of tuberculosis (TB) in the U.S. with recurrent outbreaks among the homeless and marginally housed. It has been shown for syndromic data that when exact geographic coordinates of individual patients are used as the spatial base for outbreak detection, higher detection rates and accuracy are achieved compared to when data are aggregated into administrative regions such as zip codes and census tracts. We examine the effect of varying the spatial resolution in the TB data within the San Francisco homeless population on detection sensitivity, timeliness, and the amount of historical data needed to achieve better performance measures.

**Methods and Findings:**

We apply a variation of space-time permutation scan statistic to the TB data in which a patient's location is either represented by its exact coordinates or by the centroid of its census tract. We show that the detection sensitivity and timeliness of the method generally improve when exact locations are used to identify real TB outbreaks. When outbreaks are simulated, while the detection timeliness is consistently improved when exact coordinates are used, the detection sensitivity varies depending on the size of the spatial scanning window and the number of tracts in which cases are simulated. Finally, we show that when exact locations are used, smaller amount of historical data is required for training the model.

**Conclusion:**

Systematic characterization of the spatio-temporal distribution of TB cases can widely benefit real time surveillance and guide public health investigations of TB outbreaks as to what level of spatial resolution results in improved detection sensitivity and timeliness. Trading higher spatial resolution for better performance is ultimately a tradeoff between maintaining patient confidentiality and improving public health when sharing data. Understanding such tradeoffs is critical to managing the complex interplay between public policy and public health. This study is a step forward in this direction.

## Introduction

**Figure 1:**
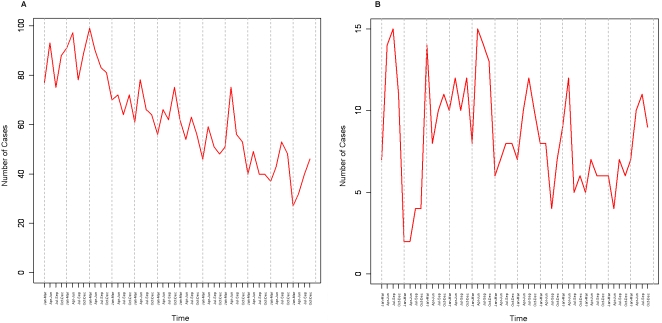
Number of TB cases in San Francisco for the years of 1991–2002: (A) general population, (B) homeless population. Each tick on the x-axis is a summation of TB cases over a three month interval. Grey dashed lines separate each year.

TB is one of the top four diseases for infection-induced mortality in the world today [1]. There are currently about 54 million people infected with the bacterium *Mycobacterium tuberculosis* with approximately 8 million new infections occurring each year. TB kills nearly 2.4 million people worldwide annually. In the U.S. alone, there are about 12.5 million people who have been infected by TB [2], with the city of San Francisco having the highest rate in the U.S. Although in recent years the incidence of TB has been declining in the San Francisco general population (see Figure 1A), it has remained relatively constant in the homeless population (see Figure 1B).

Spatial investigations of disease outbreaks seek to identify and determine the significance of spatially localized disease clusters by partitioning the underlying geographic region. The level of such regional partitioning can vary depending on the available geospatial data on cases including towns, counties, zip codes, census tracts, and exact longitude-latitude coordinates. When exact patients' locations have been used in cancer surveillance, the detection sensitivity was not appreciably higher than that obtained with larger and more conventional regional partitions such as census block groups [3], though the benefit of localized rate variation (i.e. geographic excess or shortfalls in cancer incidence for small areas) was shown in an earlier study [4]. In other works, there were few performance differences observed for larger aggregation comparisons such as block group, census tract, zip code, and town [5], [6].

More recently, Olson et al, using the method of space scan statistic [7] applied to syndromic data, has shown that when patients' exact locations are used, higher detection sensitivity is achieved as compared to center points of larger geographical regions such as zip codes and census tracts [8]. The authors demonstrated that the advantage in using higher resolution cluster detection results primarily in a reduced distortion effect that is induced by the use of large detection windows (i.e. spatial scanning windows), as compared to smaller detection windows. This problem occurs when, for example, two cases are geographically close to one another, while they reside in two separate zip codes (or census tracts). In such situations, if the geographic partitioning is by zip code (or census tract), the detection window has to be rather large to encompass both administrative regions because the cases are represented by the centroids of these regions, while a smaller detection window can capture such localized cases when the exact individual addresses are used.

In space-time surveillance of disease outbreaks, however, the interdependency between both time and space are manifested by disease clusters that are localized in time and space. Such disease localizations can be investigated through dynamic partitioning of the underlying geographic regions, where different degrees of spatial resolution can be coupled with varying levels of temporal scale to examine both the detection sensitivity and timeliness (i.e. the speed by which an outbreak is detected after it has occurred). While the benefits of using higher spatial resolutions, such as patients' individual addresses, have been examined in the context of spatial epidemiology, the spatio-temporal effects of disease localizations have not been studied under different degrees of spatial aggregation. As such, the effect of varying degrees of spatial aggregation on detection timeliness has not been investigated. At the same time, any detection method must rely on a pool of historical data to both establish a baseline of normal disease variability and estimate the model parameters. However, the amount of available data varies across surveillance programs, and historical data are often in short supply. Therefore, in addition to detection sensitivity and timeliness, the dependency on the amount of historical data must also be examined when varying spatial resolution

In this work, we use a modification of a space-time permutation scan statistic [7], [9]–[12] to examine the effect of varying degrees of spatial resolution (census tracts of patients' residences versus exact locations) on the detection sensitivity, detection timeliness, and the amount of data needed for training the model, applied to the TB data on the San Francisco homeless population for 1991–2002, using both simulated and confirmed outbreaks. This study is population-based and prospective, where historical data and documented outbreaks are used, but the detection algorithm is applied to the data as would be run in a real-time environment.

## Materials and Methods

### Data

The San Francisco Department of Public Health (SFDPH), TB Control Program (TBCP), routinely collects comprehensive information on TB cases and their contacts including demographics (e.g., age, gender, race), personal risk factors (e.g., intravenous drug use, HIV status, alcohol intake), laboratory results (e.g., skin test, chest x-ray), time of diagnosis, and primary residence addresses. For the homeless population, the geospatial information typically includes addresses fir shelters and single room occupancies (SRO-a building that houses tenants in single rooms where kitchen and bathrooms are shared for each floor). The SFDPH TBCP takes all possible means to document reported individuals infected with TB in the homeless population within San Francisco County, such that the dates of occupancies at shelters are recorded on a daily basis. This type of infrastructure enhances the SFDPH TBCP's ability to track homeless individuals to a primary residence and identify potential locations of TB transmission between individuals. As a result, the residences that are registered with the SFDPH TBCP can be used as a stable residence location for a homeless individual, given by the start and end dates recorded in their database and presented in this work.

In addition to the above data types, advances in molecular biology have made it possible to identify different bacterium fingerprints with the technique of restriction fragment length polymorphism (RFLP) and polymorphic GC-rich repetitive-sequence (PGRS) methodologies [13], [14]. With these technologies, it is possible to both identify and track specific subpopulations that have been infected with the same bacterial strain. This information can aid in outbreak investigation to identify patterns and hubs of transmission often hidden in a network of complex interactions between primary infected cases and their contacts [15], [16].

The dataset for this study consists of comprehensive information on 392 individuals that have been diagnosed by the SFDPH TBCP with active TB and identified as homeless over the time period of 1991–2002. The primary residences of these individuals were used to identify their geographical coordinates (latitudes and longitudes) using ArcGIS v9.0 (ESRI). The census tract information for identifying the tracts in which the homeless individuals reside, were obtained from generalized extracts from the Census Bureau's TIGER geographic database provided by the US Census Bureau (http://www.census.gov/geo/www/cob/index.html). There were a total of 76 unique census tracts covered by the study population. TB case data is kept electronically in a patient management database maintained by the SFDPH TBCP. All case information, including address of residence and homeless status at the time of diagnosis, was downloaded directly from the database. Census tract information was obtained from the 2000 census.

### Confirmed outbreaks— p9 cluster

During 1991–2002, an epidemic strain of TB took hold among the homeless population in San Francisco. According to the SFDPH TBCP, an outbreak is defined as 1–2 TB cases, localized in time and geographic area. Both RFLP and PGRS analyses were conducted on infected cases to identify the particular strain and associate it with previously identified molecularly similar clusters. This investigation resulted in 47 unique homeless individuals being identified as infected carriers of this previously unobserved strain, referred to as the *p9* cluster. This cluster arose at two separate time periods, with the first outbreak starting in the fall of 1995, peaking in 1996 and disappearing by the spring of 1999, with a second outbreak rapidly appearing in the fall of 2001, peaking in the summer of 2002, and disappearing in the spring of 2003. Since this cluster has now been identified as two ‘waves’ of true outbreaks, these cases were used for evaluation of the detection algorithm. More specifically, we used the dates of these confirmed *p9* outbreaks to assess both the sensitivity and timeliness of the detection algorithm presented here when using census tract centroids versus individual addresses.

### Modified space-time permutation scan statistic

**Figure 2:**
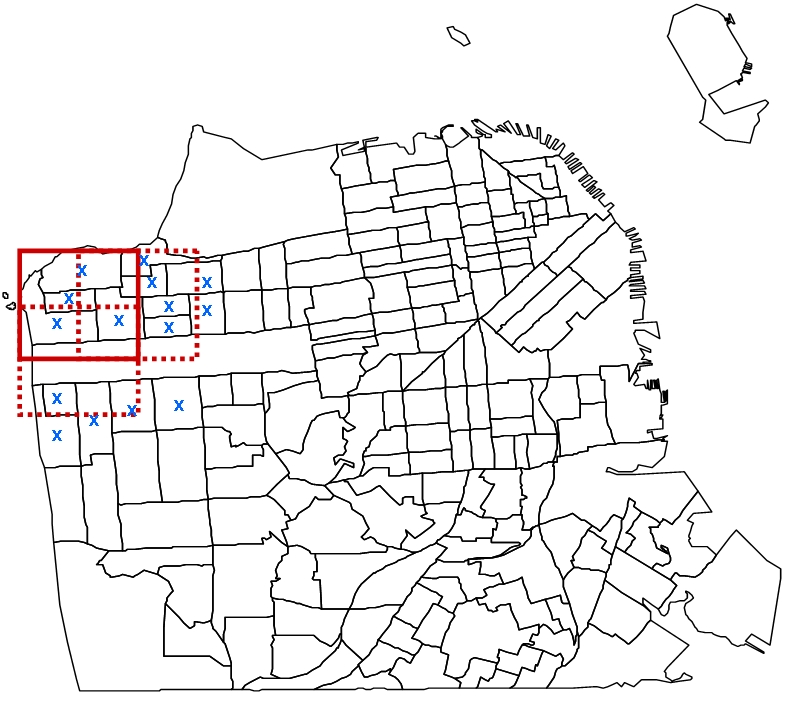
An illustration of partitioning the map of San Francisco using the overlapping square grid approach. Three overlapping squares, representing three spatial scanning windows in the detection method, are overlaid upon the northwest quadrant of San Francisco. The map is partitioned by census tracts. A single square size and only a few census tract centroids (blue ‘x’ symbols) are represented in this example for illustrative purposes.

A variation to the space-time permutation scan statistic introduced by Kulldorff et al. [10] using a square grid approach provided by Neill et al. [17] is implemented here for space-time investigation of TB outbreaks in the San Francisco homeless population. Briefly, the method can be described as follows. Instead of using circles of multiple radii as spatial bases for scanning cylinders [10], a square grid approach [17] is employed here. The method iteratively runs varied sizes of squares ranging from 0.02 km to 1 km in width, akin to varied radii sizes of circles in [10]. In every iteration of the algorithm, overlapping grids containing *p* squares, each of area *r* ^2^ are placed over the entire region, where the grid overlap is permitted at half the width of each square, representing the spatial domain. That is to say, for a particular row using a specified square size, each square is overlaid upon an adjacent square with half of the width of both squares overlapping. Then, for the next row, the same procedure is implemented, where the width of a square in the subsequent row overlaps with the adjacent square in the same row (half the width) and the square from the preceding row (half the height). Figure 2 illustrates an example of such a geographic partitioning approach using three overlapping squares overlaid on a map of the Northwest quadrant of San Francisco. The time domain, analogous to Kulldorff et al. [10], is represented by the height of such (square) cylinders. For each square, the expected number of cases, conditioned on the observed marginals is denoted by *μ* where *μ* is defined as the summation of expected number of cases in a cylinder, given by 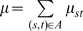 where *s* is the spatial cluster and *t* is the time span used, and 
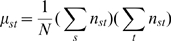
, where *N* is the total number of cases and *n_st_* is the number of cases in either the space or time window (according to the summation term). The observed number of cases for the same cylinder is denoted by *n*. It is important to note that because we do not have an accurate account of the population at risk (e.g. the size of homeless population), the expected values are derived from the TB case counts. Then the Poisson generalized likelihood ratio (GLR), which is used as a measure for a potential outbreak in the current cylinder, is given by 
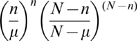
 [18]. To assign a degree of significance to the GLR value for each cylinder, Monte Carlo hypothesis testing [19] is conducted, where the observed cases are randomly shuffled, though the spatial and temporal marginals are unchanged, and the GLR value is calculated for each square [10]. This process of randomly shuffling is conducted over 999 trials and the random GLR values are ranked. A p-value for the original GLR is then assigned by its relative ranking within the random GLR values.

For our space window, we restricted the width of squares to range from 0.02 km to 1 km, while the time window was varied from 1 to 6 months, where one month is approximated by a fixed four week interval. This resulted in a total of 441 space scanning squares sampled for the census tract centroids, and a total of 4,234 for individual addresses.

For our time window, the TB case count is much lower than the daily data feeds typical of surveillance systems used to monitor emergency room visits due to the influenza-like illnesses or pharmacy sales, for example. To compensate for the smaller proportion of total cases, daily counts were aggregated into monthly (approximately 4 weeks) case counts resulting in a total of 144 data points (months). This agglomeration of cases is necessary since the notion of *early detection* of outbreaks of a chronic disease, such as TB, with long incubation period [20] requires a longer time scale. This is in contrast to syndromic surveillance of acute infectious disease (such as influenza-like illnesses), where early detection encompasses only hours to days after the start of an outbreak (see [21]–[25]) and the case counts are much greater for this shorter time period. The scan statistic is shown to handle both densities of cases. Finally, the amount of historical data used for training the model and parameter estimations varied from 1–18 months spanning the years of 1991–2002.

### Reduction of overlapping signals

Due to the number of likelihood ratios calculated, multiple testing correction becomes an important procedure for determination of significant signals. The Monte Carlo hypothesis testing step is designed to correct for this. However, there are instances where multiple signals with similar significance are detected (i.e. not having adequate power to use enough precision for distinguishing two p-values) that also share a high degree of similar information (due to the stochastic nature of process by which the p-value is calculated), such that a procedure for reduction of such redundant information is required. For example, a geographical square with a GLR of *y* that has two cases within the one month window may intersect with another square, with the same GLR value of *y* (based on the acceptable precision ) for the same time window, that is larger in size, contains three cases, and encompasses the first square. Under such circumstances when there is a 100% intersect of a smaller square with a larger square for the exact same time period and similar significance measure, a unique signal is reported by retaining the smaller square. This procedure was performed on all significant signals using the above criteria

## Results

### Confirmed outbreaks—detection sensitivity and timeliness

**Figure 3:**
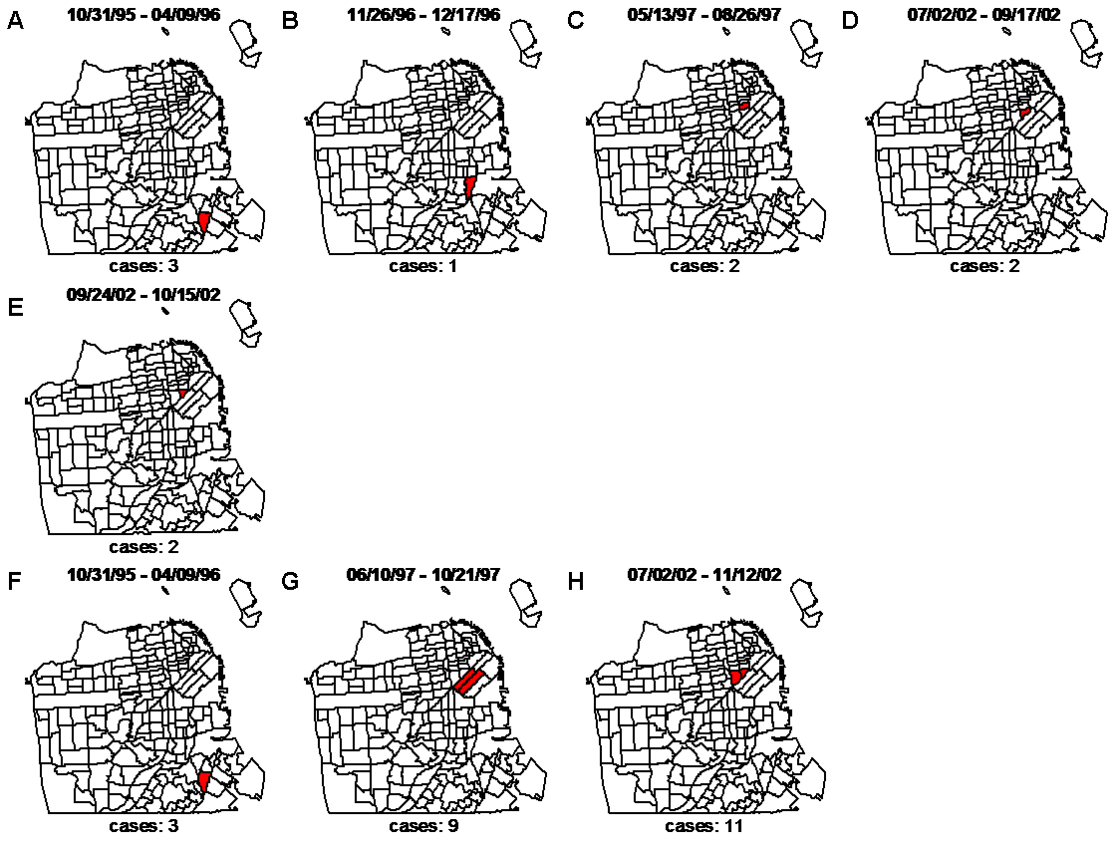
Significant signals detected by the method applied to the confirmed *p9* outbreaks using individual addresses (A–E) and census tract centroids (F–H). San Francisco map is partitioned by census tract where tracts shaded red represent the location of the significant signal detected for the specified dates in Table 1. To protect patient confidentiality, only the census tracts in plots A–E are shaded, as opposed to highlighting the exact locations.

Here we examine the detection sensitivity and timeliness of the method using the two levels of geographical resolution (i.e. census tract centroids and individual addresses) applied to the confirmed outbreaks of the *p9* cluster (Table 1 and Figure 3). As stated previously, this outbreak arose at two separate time periods: 1) 1995–1999, reaching a peak in TB infections in 1996, and 2) late in 2001–2002, reaching a peak in TB infections in the summer of 2002. To provide parity between the two methods, some assumptions were made in the case where both approaches were detecting the same signal, with overlapping, yet slightly different start and end dates. If the start or end dates for a significant signal under one geographic resolution overlapped the dates for the other, we considered them the same signal. That is, the detection method identified the same signal under both geographic constraints. The initial increase in cases that occurred in 1995, as well as the continuation of this outbreak through 1996, 1997, and the large resurgence in 2002 were detected under both spatial resolutions (see Table 1). These *p9* clusters are accurately detected at three separate time points (1995–1996, 1997, and 2002) that are consistent with the documented outbreaks of this epidemic strain. It should be noted that the two outbreaks detected in 2002 using individual addresses overlap the single outbreak detected in 2002 using census tract centroids, so this is treated as a single detection by each method.

**Table 1:** Detection timeliness and sensitivity of the method applied to the confirmed *p9* outbreaks using census tract centroids and individual addresses.

Outbreak	Individual addresses		Census tract centroids	
	Start date	End date	Start date	End date
1	10/31/1995	4/9/1996	10/31/1995	4/9/1996
2	11/26/1996	12/17/1996		
3	5/13/1997	8/26/1997	6/10/1997	10/21/1997
4	7/2/2002	9/17/2002	7/2/2002	11/12/2002
	9/24/2002	10/15/2002		

The dates detected by the space-time permutation scan statistic are in agreement with the two documented ‘waves’ of the *p9* outbreak in San Francisco County. With the exception of the first outbreak, the time windows are consistently shorter when exact coordinates are used. While the detection timeliness is greatly improved using exact coordinates, the detection sensitivity is only slightly greater using individual addresses than census tract centroids.

With exception to the significant cluster that is detected within the dates of 11/26/96–12/17/96 using individual addresses (likely indicating increased sensitivity as compared to using census tract centroids), there is not a considerable difference in the detection sensitivity of the two. One could argue that this additional cluster that was detected using individual addresses in 1996 (and not detected by census tract centroids) is evidence that supports overall better performance with individual addresses (as compared to census tract centroids), with confirmed *p9* outbreaks. By assessing sensitivity as a ratio of the number of detected (and confirmed) outbreaks to the total number of confirmed outbreaks, the use of individual addresses detects 100% of the confirmed outbreaks, whereas the use of census tract centroids detects 75% of the confirmed outbreaks. Irrespective of this last point, the detection timeliness is improved when using individual addresses. Here, the timeliness is assessed by the detection time window, which constitutes the number of weeks that are used within the height of the scanning window to calculate the expected and observed number of cases (used for the generalized likelihood ratio).

When examining the time window for similar signals under the two spatial resolutions, the detection method using individual addresses generally requires smaller time windows than that of census tract centroids. As can be seen from Table 1, the significant signal detected within the dates of 5/13/97–8/26/97 using individual addresses is detected approximately one month prior to the same outbreak that is detected using census tract centroids. In addition, the end date of this time window, using individual addresses, is approximately two months earlier. This earlier end date pattern is also demonstrated for the 2002 outbreak as well, even when the use of individual addresses identifies a significant cluster with two separate date blocks. For detection of identical outbreaks, the use of individual addresses (as compared to census tract centroids) is advantageous in reporting the significant cluster in a shorter time interval.

### Simulated outbreaks—detection sensitivity and timeliness

Given the sparse nature of the TB data on the homeless, limited number of outbreaks (as defined by the SFDPH TBCP) in the data, and nearly similar detection sensitivity measures obtained using the two spatial resolutions in the previous section, we next set out to examine the performance of the space-time detection method (previously described) to TB data infused with simulated outbreaks. By introducing simulated outbreaks into the real data, while we compensate for the relatively low TB case counts in many geographic regions, we can provide a comprehensive evaluation of detection performance and identify the inflection points in the sensitivity of the detection algorithm. Since the spread of real TB cases in some regions is rather sparse and non-uniform, we chose to implement a ‘supervised’ random dispersal of simulated cases. If numerous simulated cases are added to regions containing very few (or none) real TB cases, this can introduce a bias, or artificial case density structure that can lead to an inaccurate representation of the case spatial spread in the population at risk (i.e. TB homeless population), which would affect the clusters detected by the algorithm. To address this, the spatial structure of the real TB case spread was used as a guide when adding the random simulated cases. We applied (with supervision) a uniform addition of simulated cases to the background of real TB cases, to maintain a case density approximately consistent with the actual case structure.

Similar to the approach demonstrated in [8], the spatial scanning window size was increased from the smallest size of 0.02 km to the largest size of 1 km, while keeping the time window fixed, since our first interest was to establish the difference in detection sensitivity between census tract centroids and individual addresses based on a spatial resolution. A geographic region was first chosen that contained five real TB cases within a 0.2 km range. The simulated points were distributed in multiple adjacent administrative regions (census tracts) ranging from 1 to 4. A total of six simulated cases were then randomly placed in one administrative region and the percentage of significant clusters detected was calculated as the amount of cases (i.e. simulated and actual) detected divided by the total number of cases in the one administrative region. These six simulated cases were then split into two adjacent administrative regions, each containing three simulated cases (in addition to the actual cases) and the same calculation was performed. Next, the six simulated cases were split into three administrative regions, where two cases each were partitioned into three separate administrative regions (with actual cases), and the calculation was performed again. Finally, the six cases were assigned to four separate administrative regions (two regions containing two cases and two regions containing one case each, along with the actual cases), and the calculation was performed a final time. This approach was conducted a total of 12 separate times, starting each iteration with a new region of size 0.2 km, containing approximately five total real TB cases. Then the average percentage of significant clusters detected across all 12 iterations was calculated. This entire process was conducted first with a spatial scanning window using census tract centroids, then again with a spatial scanning window using individual addresses to compare the detection sensitivity between the two, while keeping the time window fixed.

**Figure 4:**
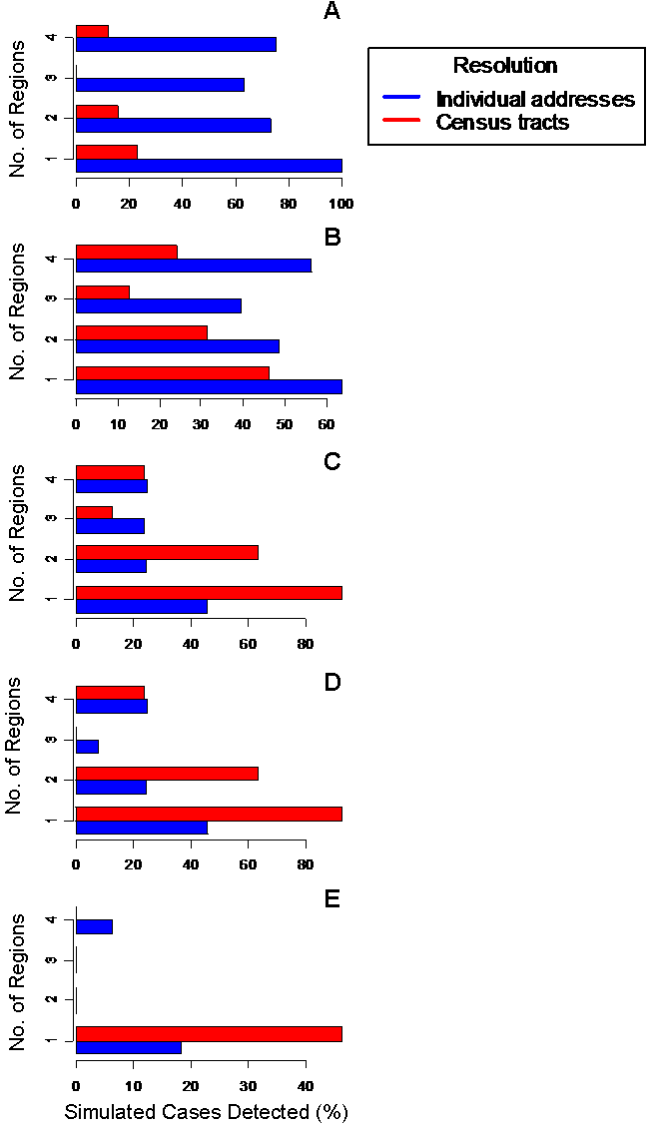
Average detection sensitivity of the method applied to simulated outbreaks. Cases were simulated in one to four adjacent census tracts using a fixed time window with increasing spatial window. The percentage of outbreaks detected are reported using individual addresses (blue bars) and census tract centroids (red bars) for spatial window sizes of (A) 0.02 km, (B) 0.1 km, (C) 0.2 km, (D) 0.5 km, and (E) 1 km, where in each case, simulated outbreaks were added to increasing number of census tracts (1 to 4) . This simulation was conducted on 12 separate geographic regions of 0.2 km in range and the average detection percentage was calculated. For each plot, the horizontal axis is scaled to the maximum detection sensitivity percentage to better visualize the primary comparison between individual addresses and census tract.

When the scanning window is small, the method attains greater detection sensitivity using individual addresses regardless of the number of regions within which the cases are added (see Figure 4A–B). As the scanning window size is set to 0.2 km (Figure 4C) and increased to larger values (Figure 4C–E), this trend continues only for the cases distributed over the multiple administrative regions (i.e. 3 and 4). The census tract centroids have greater detection sensitivity when cases are distributed over fewer administrative regions (1 and 2) using scanning window sizes of 0.2 km and greater. At the largest scanning window size (1 km), there are very few cases detected using either of the spatial resolutions, due to the convergence of the cluster grid and overall detection region.

The speed of detection (i.e. detection timeliness) is one of the most important performance measures for disease surveillance. In the space-time permutation scan statistic, the time window is varied at time spans ranging from one month to six months and anchored on the last month of the time window, using data restricted up to that point. This methodology provides a pseudo-real time scan, prospectively, where it is assumed that data is only available up to the point of running the space-time permutation scan statistic. Outbreaks that occur in a short time interval for a specific geographic region or adjacent regions are detected within the small time window size, whereas, those outbreaks that have more sparse case counts with time (i.e. spread out over a longer time span than 1–2 months) for a specific geographic region or adjacent regions, are detected within a larger time window size. In the context of a disease such as TB with chronic characteristics, we denote that an outbreak is detected early if it is identified within months (less than a year) from the start of the outbreak. To assess the differential detection timeliness of the method using individual addresses and census tract centroids, additional cases were simulated (similar to how this was conducted before), where this time, the spatial window was fixed at 0.2 km and the time window was increased from 1–6 months, anchoring the scanning window on the last month of the scan date. The spatial window of size 0.2 km was selected because this window size gave similar sensitivity results using both individual addresses and census tract centroids in the spread across four administrative regions, for the fixed time analysis example (examined above). Using the same conditions, geographic regions, and the same frequency of simulated cases as above, the approach was repeated, though for this analysis, the simulated cases were added over a time span of six months, one case for each month. This process was repeated 12 times, choosing the same starting geographic regions as were selected in the fixed time simulation method above, and the signal p-values (calculated from the Monte Carlo hypothesis test) were recorded for the administrative regions that had successive simulated cases added at each time window. Then the average p-value was calculated across the 12 simulations and these values were transformed (-log), as is often done to give a more linear measure for comparison between values. This approach (where the time window is varied and the spatial window is fixed) was used to evaluate the detection timeliness of the method between using individual addresses and census tract centroids.

**Figure 5:**
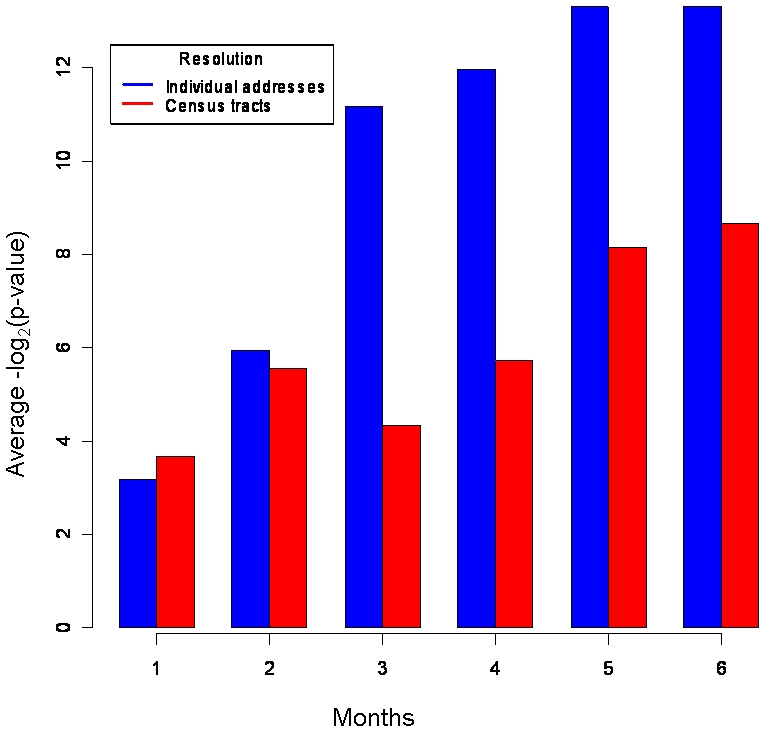
Average detection timeliness of the method applied to simulated outbreaks using a fixed spatial window of 0.2 km with increasing time window. The average -log_2_ transformed p-value was calculated across the same 12 geographic regions as those used in Figure 4 for individual addresses (blue bars) versus census tract centroids (red bars) with increasing counts of simulated cases, one for each month.

As can be seen in Figure 5, at increasing time windows greater than two months, more significant clusters are detected with the use of individual addresses. At time windows less than three months, no noticeable difference is observed between the two methods. Both individual addresses and census tract centroids demonstrate a linear relationship with time after two months. So when using a fixed space window and increasing the time domain, the method, using individual address, detects more significant clusters at a time window greater than two months.

### Historical data requirement

**Figure 6:**
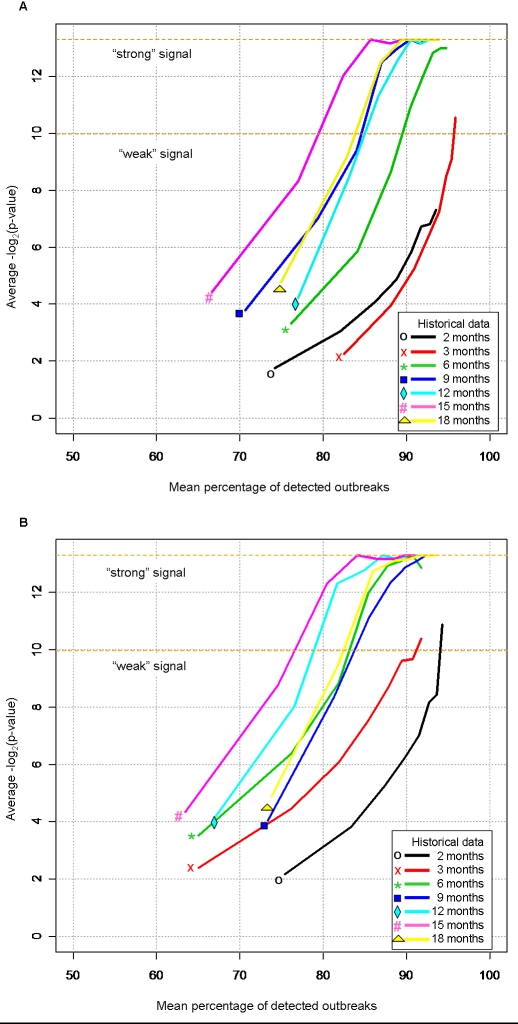
Sensitivity of detection of simulated outbreaks with varied amounts of historical data using (A) census tract centroids and (B) individual addresses. To determine the significance of detected simulated outbreaks with varying amount of historical data a fixed time window of one month was used. The orange dashed lines represent what the authors designate as significant “weak” and “strong” signals corresponding to p-values of 0.001 and 0.0001, respectively.

The accuracy of surveillance algorithms is often determined by assessments on retrospective data. Documented outbreaks in the past are used as ground truth to measure model performance. Depending on the algorithm, the larger the pool of historical data, the more reliable are the performance measures, since the assessment of normal variability improves with measurements over time We assessed the accuracy of the algorithm using the two spatial resolutions by simulating increasing cases on a background of real TB cases, using varying amounts of historical data. First, we randomly selected a geographic region, and simulated 1–8 cases using 4 weeks (approximately 1 month) of historical data. Then we did this again increasing the amount of historical data from 4 weeks to 72 weeks (approximately 18 months), evaluated at four week intervals. The percentage of simulated cases with the background of real cases in the scan window was then recorded, as well as the detection p-value, calculated from the Monte Carlo hypothesis test, for that scan window. For example, if three cases are simulated within a background of three cases, this region has had a 100% (3/3) increase in cases, 50% (3/6) consisting of simulated cases, and the detection p-value calculated may be 0.20. Now if three more simulated cases are added to the previous three simulated cases and the background of three cases, this region now has had a 200% (6/3) increase in cases, 67% (6/9) consisting of simulated cases, and the detection p-value calculated may be 0.01. We implemented this methodology using the p-values (Figures 6A and 6B y-axis) and simulated case percentages (Figures 6A and 6B: x-axis), for census tract centroids (Figure 6A) and individual addresses (Figure 6B) starting with only one month of historical data to draw upon, all the way up to 18 months. Then this procedure was repeated 1,000 times for different randomly selected regions.

We conducted this evaluation by fixing the spatial window at 0.2 km and repeatedly increasing the height of the cylinder (time window) from one to six months, while for each time window the amount of historical data was increased from one to 18 months. Figures 6A and 6B illustrate the respective sensitivity of detection method using census tracts and exact coordinates to the amount of historical data where the time window was fixed at one month. The results for time windows of 2–6 months were similar to those of the one month window, so we have only reported the one month window results in Figure 6.

Figures 6A and 6B illustrate the sensitivity of significant signals to the amount of historical data required to detect outbreaks within a one-month window. The orange dashed lines represent what we designate as a significant “weak” and “strong” signal, corresponding to p-values of 0.001 and 0.0001, respectively. Though both of these p-value thresholds are considered to indicate a significant signal, we find that there are advantages for this example in using both thresholds. When limiting the historical data to small quantities, no significant clusters are detected at the “strong” signal threshold (i.e. p-value<0.0001), whereas at the lower “weak” signal threshold, the use of all intervals of historical data provide significant clusters detected. On the other hand, when only using the “weak” signal threshold, the point of saturation (i.e. maximization of cluster detection scores) is not observed. As a result, we find that both p-value thresholds can be considered significant in this simulation example for cluster detection, with the difference in threshold values providing different comparison information.

When the availability of historical data is limited to only 2–3 months, using individual addresses provide a more sensitive measure than census tracts. With the use of census tracts, there is neither a “weak” nor “strong” significant signal detected with two months of historical data, as opposed to the “weak” signal observed for individual addresses. In addition, with three months of historical data, the use of individual addresses demonstrate detection of a “weak” signal when simulated cases were added at 90%, whereas, the use of census tracts allows detection of the “weak” signal, requiring simulated cases to be added at 95%. When the availability of historical data is limited to six months, the method detects a “weak” signal at 90% of simulated cases using census tracts, while it detects a “strong” signal at about the same 90% using individual addresses. Note that increased detection sensitivity is implied when the detection method identifies fewer simulated cases using smaller amounts of historical data under individual addresses.

## Discussion

We investigated the effect of varying the spatial resolution in a variant of a widely used space-time detection technique on the sensitivity and timeliness of identifying both simulated and confirmed TB outbreaks, and examined the dependency of these performance measures on the amount of historical data required. We showed that when exact patients' locations are used and the method was applied to real TB outbreaks, both the detection sensitivity and timeliness are improved and smaller amount of historical data is required compared to when census tracts are used as the spatial base for geographic partitioning. When outbreaks are simulated, while the detection timeliness is consistently improved and smaller amount of historical data is required for training the model, depending on the size of the spatial scanning window (squares) the detection sensitivity varies. Specifically, when the size of the scanning window is sufficiently large and the simulated cases are placed into one or two census tracts, the detection method identifies more outbreaks using census tracts. Such a dichotomy in detection sensitivity and the results of Figure 4 warrant some discussion.

First, when individual patients' coordinates are used as basis for geographic partitioning, the detection method consistently performs better, irrespective of the number of census tracts to which the simulated cases are assigned, if the size of the scanning window is sufficiently small (<0.2 km). Second, when the size of the spatial scanner is sufficiently large (0.2 km or larger) and the simulated cases are assigned to one or two census tracts, then the method performs better under census tract-based partitioning. Third, when the size of the spatial scanner is sufficiently large (0.2 km or larger) and the simulated cases are placed in more than two census tracts (three or four), then the detection sensitivity is better using individual coordinates and further enlarging of the width of the spatial scanning window does not result in better performance using census tracts (see Figure 4).

When simulating cases for the sensitivity comparison, the real TB cases were infused with simulated cases distributed (in a ‘supervised’ random fashion) within 1–4 census tracts. This was conducted in such a manner as to maintain the underlying distribution of the real TB cases and not bias the spread of cases around any particular region. While the observed dichotomy in detection sensitivity when outbreaks were simulated should be systematically studied in future works, insights gained from this study as to how the distribution of cases may impact the detection performance, and thus the choice of spatial resolution, can be shared here. To approximately measure the disease case-spread with respect to each other as well as with respect to the center of tracts, under both simulated and confirmed outbreaks, we estimated the mean and standard deviation (SD) for two distributions: (1) distribution of distance between cases and the center of their census tracts, and (2) pairwise distance distribution of cases. The first set of statistics gives an indication of the extent of case-spread with respect to the center of their census tracts while the second is an indication of the extent of case spread with respect to each other. Qualitatively, if cases on average are closer to the center of their census tracts than to each other, then presenting each patient by the census tract center should introduce minimal distortion in the actual case distribution, compared to when they are further, which then depending on the SD values, the detection method may perform similarly under the two spatial resolutions.

When data were infused with simulated cases, the statistics for the first distribution were mean = 0.32 km and SD = 0.60, and for the second distribution, mean = 0.45 km and SD = 0.38. For the confirmed outbreaks, the statistics for the first distribution were mean = 0.15 km and SD = 0.20, and for the second distribution, mean = 0.29 km and SD = 0.20. In both cases, the mean for the first distribution is smaller than that of the second, which implies that on average, cases are closer to the center of their census tracts than to each other. At the same time, under simulated outbreaks, the SD value is larger for the first distribution than for the second. A larger SD for the first distribution under simulated outbreaks implies that there are areas (census tracts) in the region that may contain very few or no cases, while there are other tracts with more cases, and thus some level of clustering in the latter tracts exist. Therefore, it is no surprise that the method performs better using census tracts when a fixed number of simulated cases are added to one or two census tracts, amplifying the existing clustering effect, and when the spatial base is sufficiently large to encompass such clusters. Under confirmed outbreaks, a smaller mean value for the first distribution is accompanied with a value of SD that is the same for the second distribution, implying similar clustering effect under the two distributions. This is perhaps why the detection sensitivity was not significantly improved when exact coordinates were used and the method was applied to sparse and small numbers of confirmed outbreaks (see Table 1).

To summarize, while using exact patients' coordinates, the detection method almost invariably was able to identify localized clusters of smaller sizes earlier in time, which is a critical property of real time surveillance for timely containment of disease outbreaks. A larger improvement in sensitivity was obtained when the cases were randomly generated. This is a sensible result, because the real TB case distribution in the event of an actual outbreak is not expected to characterize a uniform distribution. Factors affecting the spread and transmission of TB in the homeless population, such as localization of shelters and SROs to specific geographic regions, and the high prevalence of intravenous drug use and HIV among the homeless, result in spatial clusters of TB that are topologically different from those attained under a random distribution of cases in space. Thus, we infer that systematic characterization of the extent and topology of disease case-spread derived from historical data can widely benefit real time surveillance and guide public health investigations with respect to detection and control of infectious diseases. While the decision on which spatial resolution results in improved detection sensitivity may depend on localization properties of historical case spread, we showed that the detection timeliness is consistently improved when the detection method uses patients' coordinates as the spatial base for search.

Finally, trading higher spatial resolution for increased performance is ultimately a tradeoff between maintaining patient confidentiality and improving public health. While these features are critical to real time surveillance, maintaining patient confidentiality introduces a challenge to the timely investigation of outbreaks. The complex interplay between public policy and public health may be better managed by understanding and balancing the associated risks in each problem domain. As critical as this topic of debate may be, it is outside the scope of this work.
